# Supplementary material for: Cryo-EM structure of human O-GlcNAcylation enzyme pair OGT-OGA complex
Source: Nat Commun. 2023 Oct 31;14:6952. doi: 10.1038/s41467-023-42427-8 (PMC10618255; doi:10.1038/s41467-023-42427-8)
Supplement: Supplementary file 1 — Supplementary Information [file 41467_2023_42427_MOESM1_ESM.pdf]

Supplementary Data and Figures for

Cryo-EM structure of human O-GlcNAcylation enzyme pair OGT-OGA complex

Ping Lu[1,2,3,#], Yusong Liu[2,3,4,#], Maozhou He[2,3], Ting Cao[2,3], Mengquan Yang[1,2,3], Shutao Qi[2,3], Hongtao Yu[2,3,*], Haishan Gao[2,3,*]

[1]College of Life Sciences, Zhejiang University, Hangzhou, Zhejiang, China

[2]New Cornerstone Science Laboratory, School of Life Sciences, Westlake University, Hangzhou, Zhejiang, China

[3]Westlake Laboratory of Life Sciences and Biomedicine, Hangzhou, Zhejiang, China

[4]School of Life Sciences, Fudan University, Shanghai, China

[#]These authors contributed equally to this work

[*]Correspondence: yuhongtao@westlake.edu.cn or gaohaishan@westlake.edu.cn

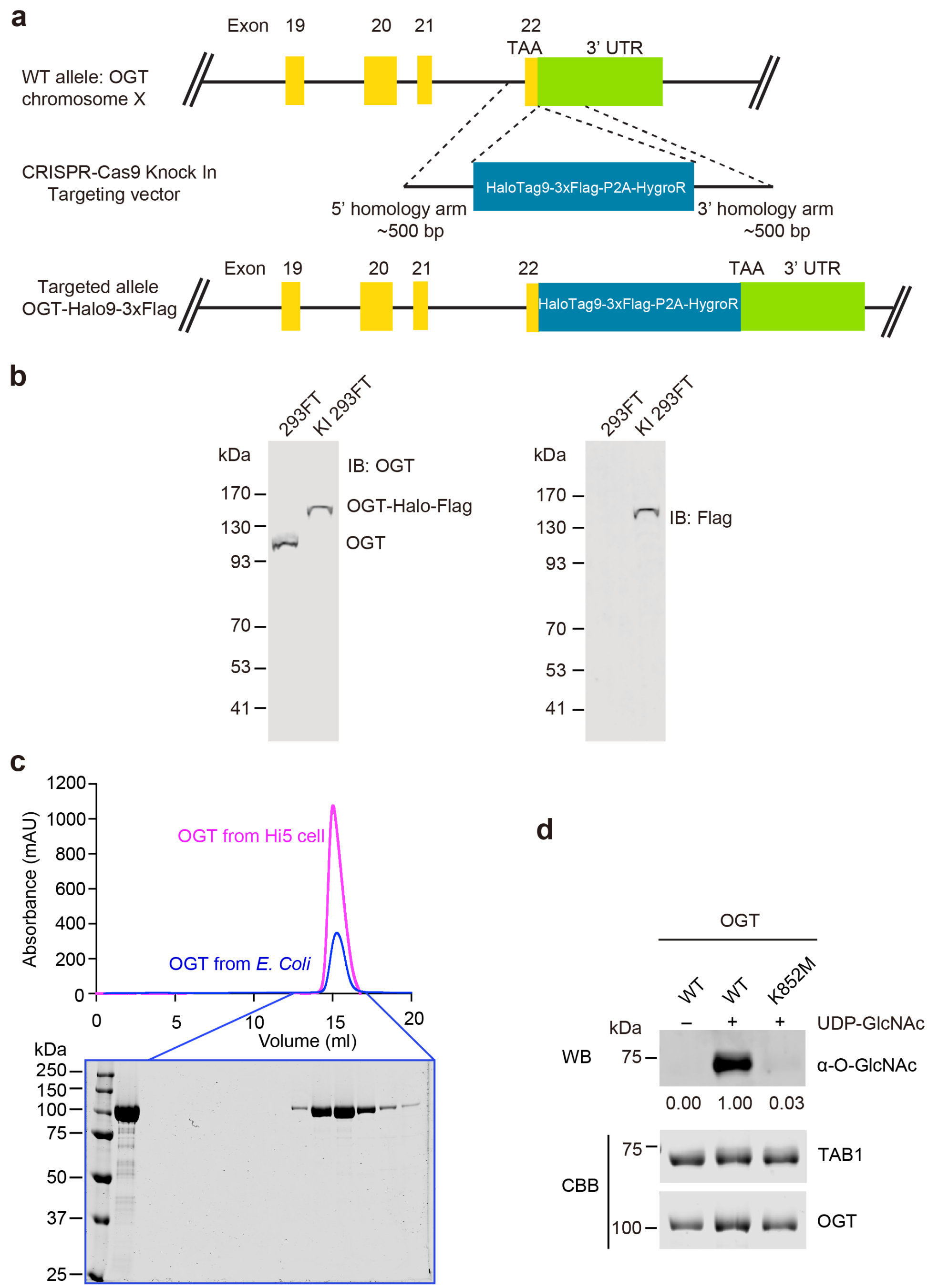

Supplementary Fig. 1 CRISPR-Cas9 editing and characterization for human OGT. a, CRISPR-Cas9 knock-in strategy used for human OGT in the current study. **b,** Confirmation of the successfully edited OGT gene by Western blot. Left, 293FT cells lysate were blotted with OGT antibody (Abcam, ab177941); Right, 293FT cells lysate were blotted with Flag antibody (Sigma-Aldrich, F1804). **c**, Gel filtration profiles of human OGT protein expressed and purified from insect cells (magenta curve) and *E. coli* (blue curve). **d**, O-GlcNAcylation assay of TAB1 with recombinant OGT wild type (WT) or its catalytically inactive K852M mutant. The relative O-GlcNAc levels were quantified and indicated below. WB, Western Blot; CBB, Coomassie

Brilliant Blue. The experiment was repeated thrice with similar results. Source data are provided as a Source Data file.

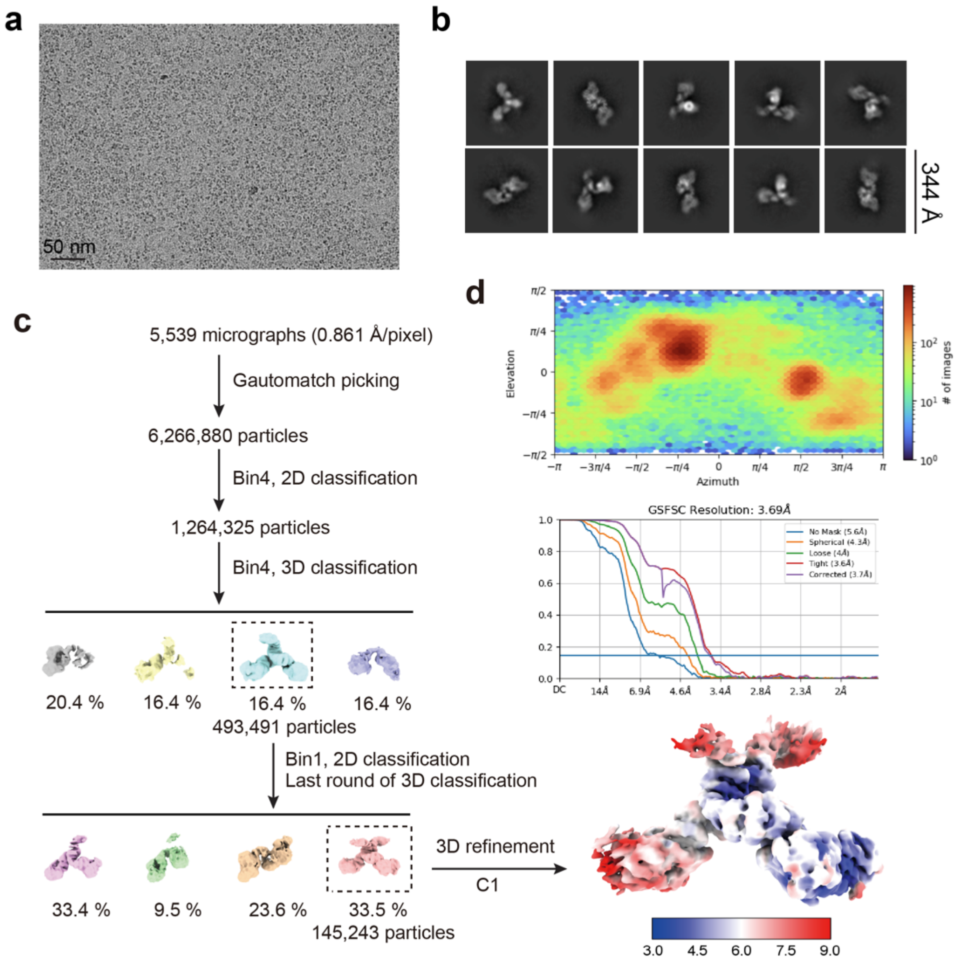

Supplementary Fig. 2 Cryo-EM analysis of human OGT homodimer. a, Representative Cryo-EM micrograph of human OGT. **b**, Representative 2D class averages of human OGT. **c**, Cryo-EM image processing flowchart for human OGT homodimer, with the final EM map colored by local resolution. **d**, The angular distribution of particles used in the final reconstruction and the Gold-standard FSC curve of the final EM map for the OGT homodimer.

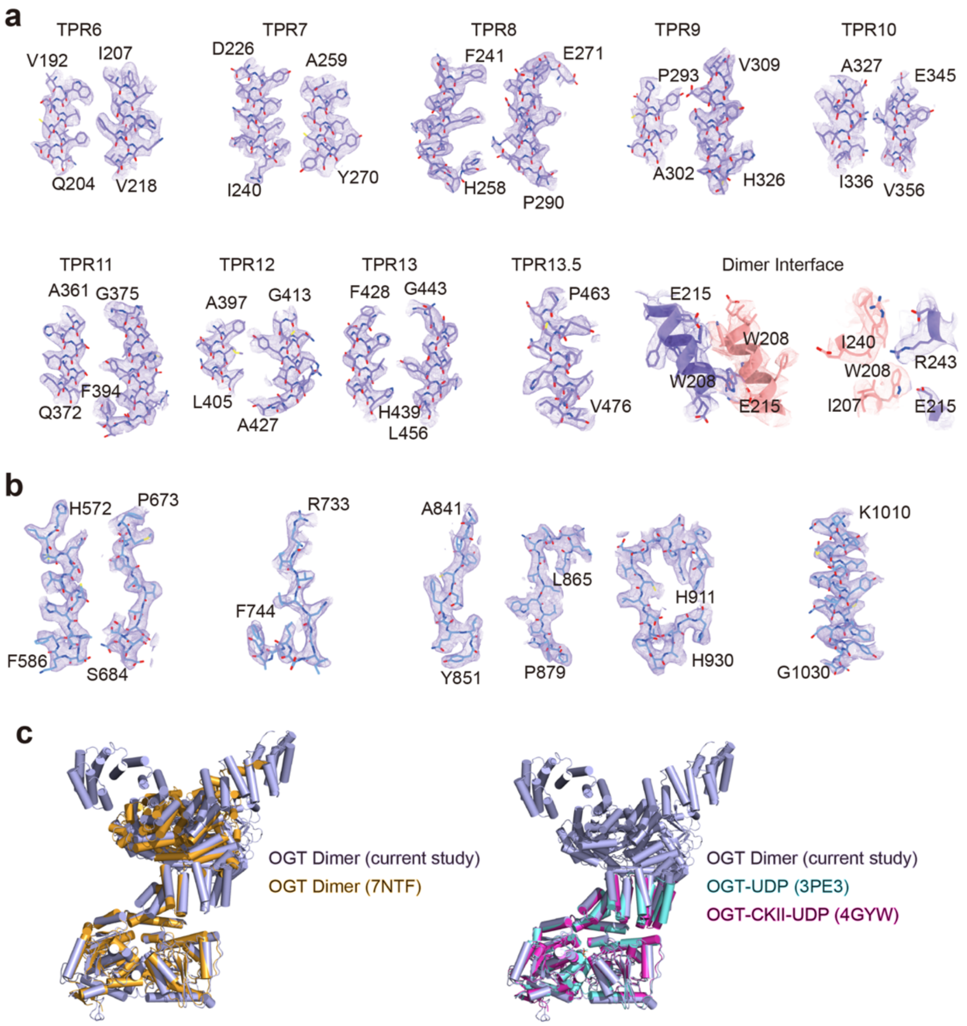

Supplementary Fig. 3 Representative cryo-EM density maps of key structural elements of human OGT. a, Local density maps of TPRs and the dimerization interface for human OGT. **b**, Local density maps of selected structural elements of human OGT GTD. **c,** Comparison for OGT dimer in the current study and previously solved OGT structures. Left, RMSD =1.085 Å for the backbone C-alpha atoms in the catalytic domain $GTD_{477\text{-}1046}$ of OGT; Right, RMSD =1.309 Å, 1.280 Å, respectively, for the backbone C-alpha atoms in the catalytic domain $GTD_{477\text{-}1046}$ of OGT.

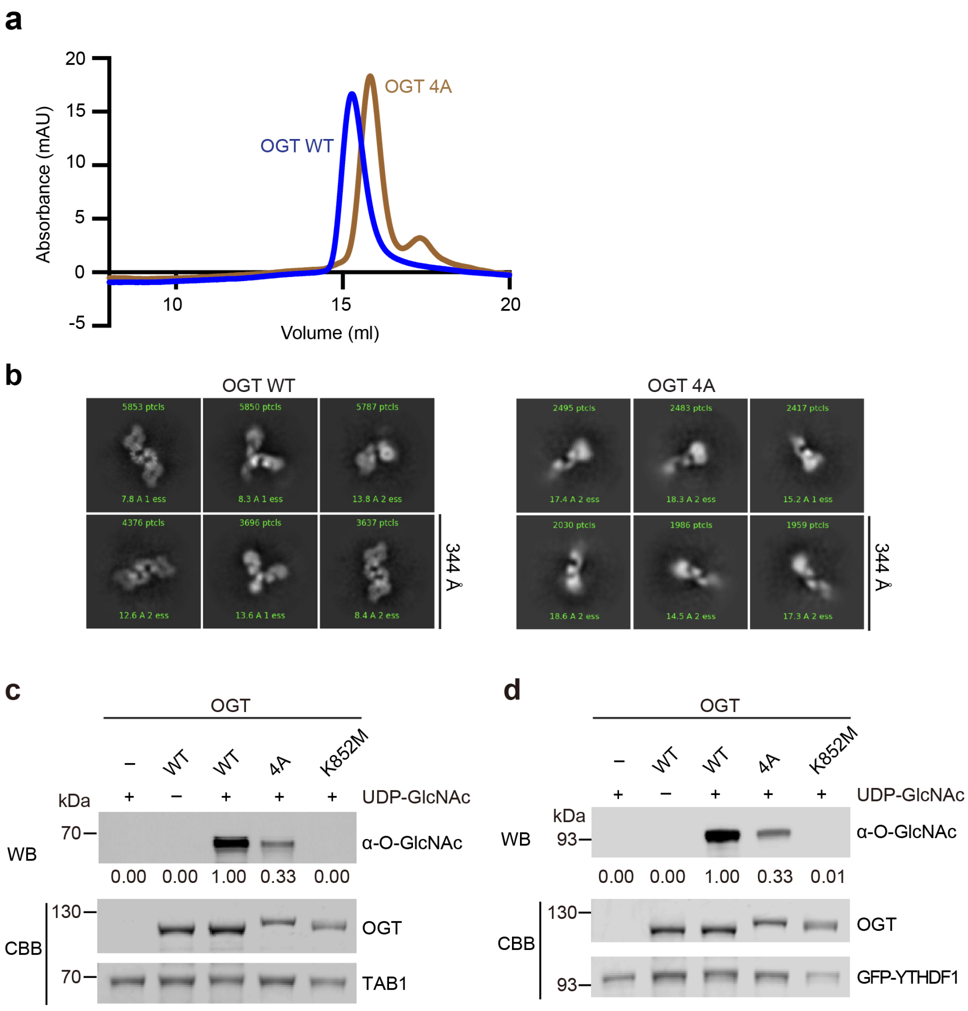

Supplementary Fig. 4 Characterization of human OGT and its 4A mutant. a, Gel filtration profiles of human full-length OGT wild type (WT; blue curve) and 4A mutant (brown curve). **b**, Representative 2D class averages of OGT WT (left panel) and 4A mutant (right panel). **c,** O-GlcNAcylation assay of TAB1 with different OGT proteins. **d,** O-GlcNAcylation assay of YTHDF1 with different OGT proteins. The relative O-GlcNAc levels were quantified and indicated below the gels in **c** and **d**. The experiment was repeated thrice with similar results for **c** and **d**. Source data are provided as a Source Data file.

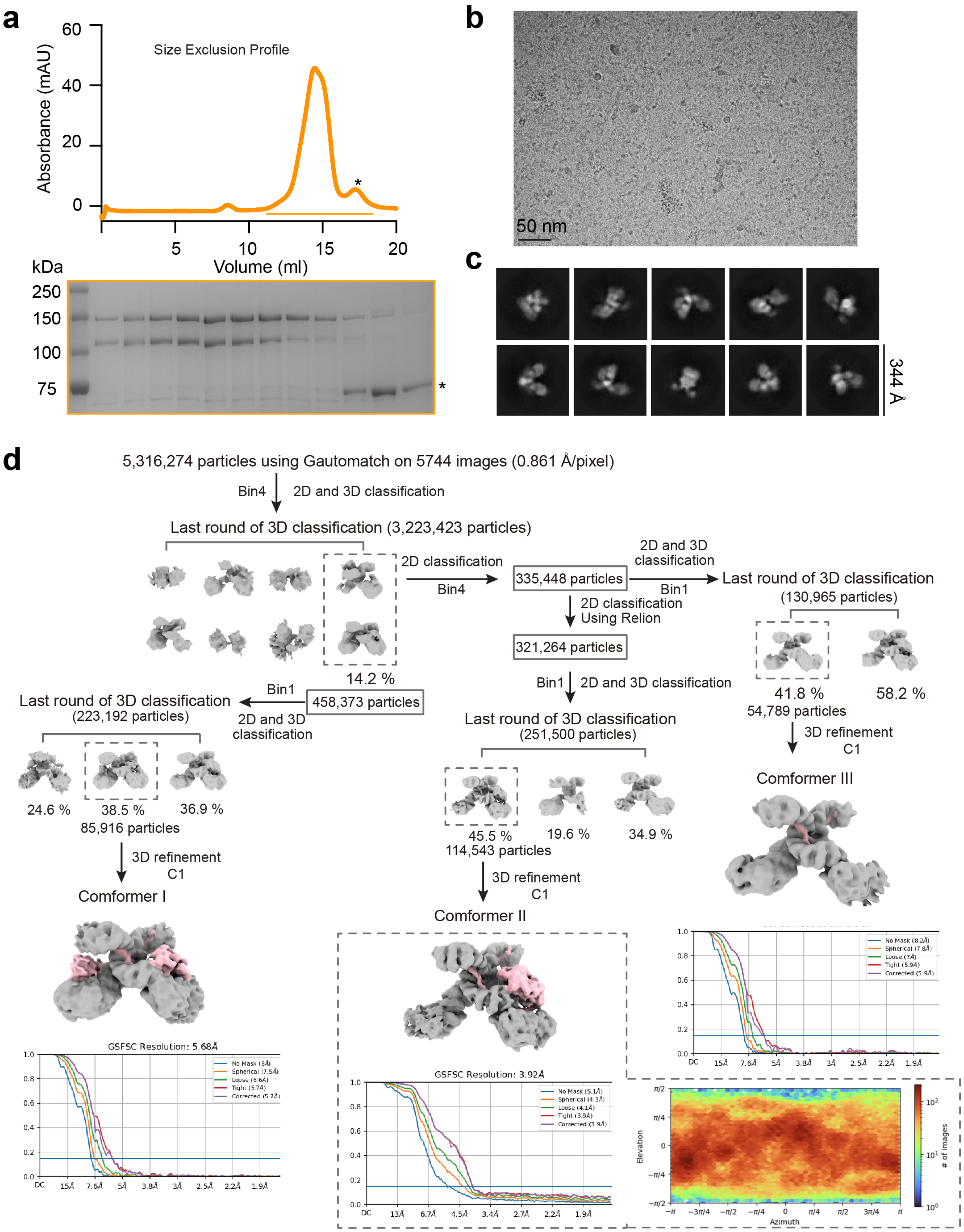

Supplementary Fig. 5 Cryo-EM analysis of human OGT-OGA complex. **a**, Gel filtration profile of human OGT-OGA complex expressed and purified from *E. coli*. Coomassie-stained

gel of protein-containing fractions is shown below. The asterisk indicates a contaminating protein. **b**, Representative cryo-EM micrograph of the OGT-OGA complex. **c**, Representative 2D class averages of the OGT-OGA complex. **d**, Cryo-EM data processing flowchart of human OGT-OGA complex. The three major classes (Conformer I, II, and III) of human OGT-OGA complex are shown. Volumes of OGT and OGA are colored in gray and red, respectively. Source data are provided as a Source Data file.

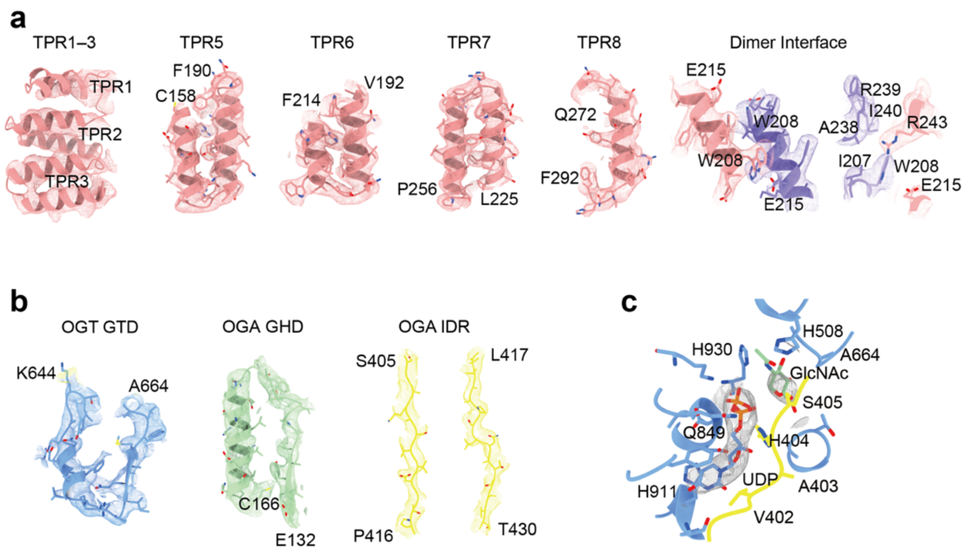

Supplementary Fig. 6 Representative cryo-EM density maps of key structural elements of the OGT-OGA complex. a, Local density maps of TPRs and the dimer interface of the OGT-OGA complex. **b**, Local density maps of OGT GTD and the GHD and IDR of OGA. **c,** Local density maps of UDP and glycosylated S405 of OGA.

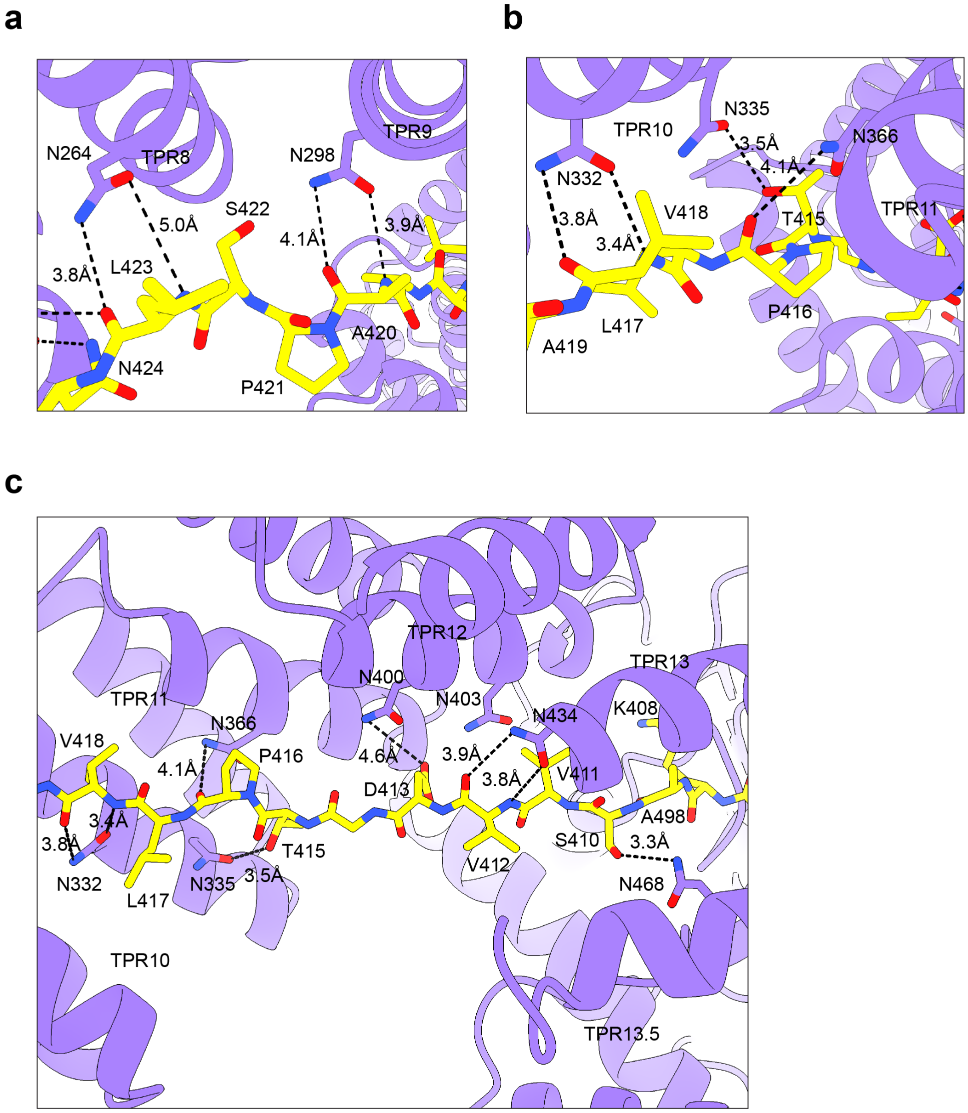

Supplementary Fig. 7 Interactions between OGT and the flexible region of OGA. a, Close-up view of OGT TPR 8-9 units (colored in purple) interacting with OGA (colored in yellow). **b**, Close-up view of OGT TPR 10-11 interacting with OGA. **c**, Close-up view of OGT proximal units (10-13) interacting with OGA.

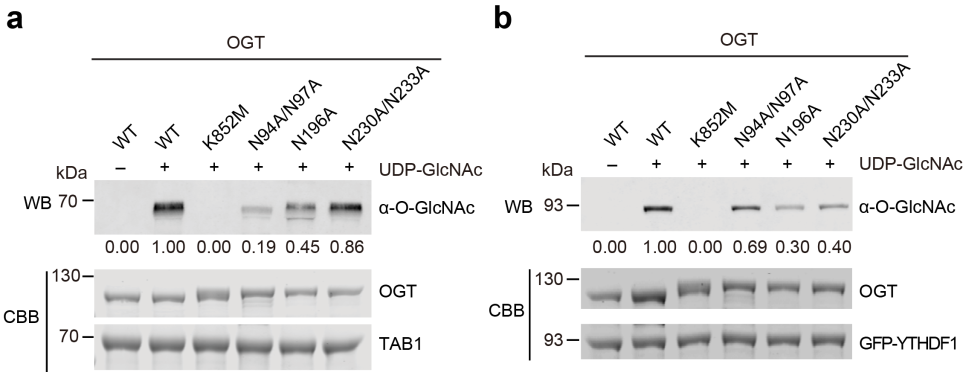

Supplementary Fig. 8 The hotspots of critical residues in human OGT TPR for substrates modifications. **a**, O-GlcNAcylation assay of TAB1 with different OGT proteins. **b,** O-GlcNAcylation assay of YTHDF1 with different OGT proteins. The relative O-GlcNAc levels were quantified and indicated below the gels in **a** and **b**. The experiment was repeated thrice with similar results for **a** and **b**. Source data are provided as a Source Data file.

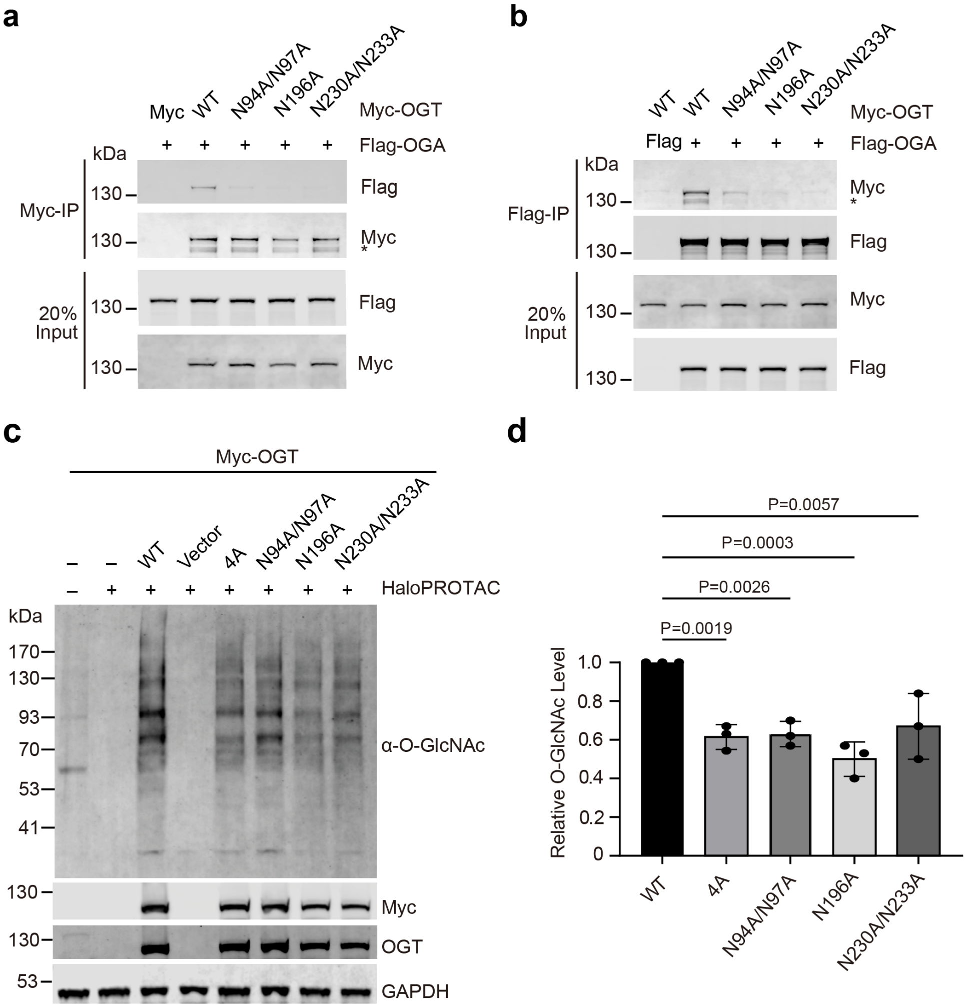

Supplementary Fig. 9 Co-Immunoprecipitation assay between OGA and different OGT mutants in human cells. a, Immunoprecipitation assay using Myc-tagged OGT mutants to pulldown Flag-tagged OGA. **b,** Immunoprecipitation assay using Flag-tagged OGA to pulldown Myc-tagged OGT mutants. **c,** 293 cells with the endogenous OGT loci tagged with HaloTag were treated with or without HaloPROTAC and transfected with the indicated Myc-OGT plasmids. Total cell lysates were blotted with the indicated antibodies. **d**, Quantification of the relative cellular O-GlcNAc levels in **c**. Mean ± SEM (n = 3 independent experiments, One-way ANOVA was used to calculate the p values). The experiment was repeated thrice with similar results for **a**, **b**, and **c**. Source data are provided as a Source Data file.

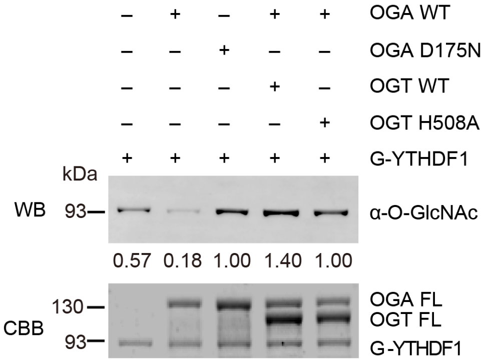

Supplementary Fig. 10 O-GlcNAcylation removal assay of glycosylated YTHDF1 by OGA and OGT-OGA complex. O-GlcNAcylated YTHDF1 (G-YTHDF1) was incubated with OGA or the catalytically inactive OGA D175N mutant in the absence or presence of the indicated OGT proteins. The reaction mixtures were analyzed by SDS-PAGE, stained with Coomassie brilliant blue (CBB), and blotted with the anti-O-GlcNAc antibody (RL2). The relative O-GlcNAc levels were quantified and indicated below. The experiment was repeated thrice with similar results. Source data are provided as a Source Data file.

Supplementary Table 1 Cryo-EM data collection, refinement, and validation statistics

	OGT	The OGT–OGA complex		
		II	I	III
**Data Collection and Processing**				
Microscope	FEI Titan Krios			
Magnification	105,000			
Voltage	300			
Electron dose (e^{-}/Å^{2})	50			
Detector	Gatan K3 Summit			
Defocus range (um)	-1.8 to -2.3			
Pixel size (Å/pixel)	0.861			
Micrographs (no.)	5,539	5,744		
Initial particles (no.)	6,266,880	5,316,274		
Final particles (no.)	145,243	114,543	85,916	54,789
Symmetry imposed	C1	C1		
Map resolution (Å)	3.69	3.92	5.68	5.86
FSC threshold	0.143	0.143	0.143	0.143
**Model composition**				
Protein residues	2034	2419	/	/
Ligands	0	2		
**Refinement**				
Initial model used (PDB code)	1W3B and 3PE3	1W3B, 3PE3 and 5UN9		
Model resolution (Å)	3.7	3.9		
FSC threshold	0.143	0.143		
Map sharpening B factor (Å^{2})	-137	-146		
**Validation**				
MolProbility score	2.21	2.96		
EMRinger score	1.27	0.84		
Clash score	18.12	28.43		
Rotamer outliers (%)	0.00	5.09		
C_{β} outliers (%)	0.00	0.00		
**R.m.s deviations**				
Bonds length (Å)	0.006	0.006		
Bonds Angle (°)	0.840	0.992		
**Ramachandran plot (%)**				
Favored	92.96	92.24		
Allowed	7.04	7.76		
Outliers	0.00	0.00		
**Deposition**				
PDB ID	7YEA	7YEH		
EMDB ID	EMD-33768	EMD-33773	EMD-33767	EMD-33769
