# Supplementary material for: Cryo-EM structure of human O-GlcNAcylation enzyme pair OGT-OGA complex
Source: Nat Commun. 2023 Oct 31;14:6952. doi: 10.1038/s41467-023-42427-8 (PMC10618255; doi:10.1038/s41467-023-42427-8)
Supplement: Supplementary file 3 — Reporting Summary [file 41467_2023_42427_MOESM3_ESM.pdf]

nature portfolio

Corresponding author(s): Hongtao Yu, Haishan Gao

Last updated by author(s): Sep 25, 2023

Reporting Summary

Nature Portfolio wishes to improve the reproducibility of the work that we publish. This form provides structure for consistency and transparency in reporting. For further information on Nature Portfolio policies, see our Editorial Policies and the Editorial Policy Checklist.

Statistics

For all statistical analyses, confirm that the following items are present in the figure legend, table legend, main text, or Methods section.

n/a	Confirmed	
☐	☒	The exact sample size (*n*) for each experimental group/condition, given as a discrete number and unit of measurement
☐	☒	A statement on whether measurements were taken from distinct samples or whether the same sample was measured repeatedly
☐	☒	The statistical test(s) used AND whether they are one- or two-sided<br>*Only common tests should be described solely by name; describe more complex techniques in the Methods section.*
☒	☐	A description of all covariates tested
☒	☐	A description of any assumptions or corrections, such as tests of normality and adjustment for multiple comparisons
☐	☒	A full description of the statistical parameters including central tendency (e.g. means) or other basic estimates (e.g. regression coefficient) AND variation (e.g. standard deviation) or associated estimates of uncertainty (e.g. confidence intervals)
☒	☐	For null hypothesis testing, the test statistic (e.g. *F*, *t*, *r*) with confidence intervals, effect sizes, degrees of freedom and *P* value noted<br>*Give P values as exact values whenever suitable.*
☒	☐	For Bayesian analysis, information on the choice of priors and Markov chain Monte Carlo settings
☒	☐	For hierarchical and complex designs, identification of the appropriate level for tests and full reporting of outcomes
☒	☐	Estimates of effect sizes (e.g. Cohen's *d*, Pearson's *r*), indicating how they were calculated

Our web collection on statistics for biologists contains articles on many of the points above.

Software and code

Policy information about availability of computer code

Data collection: AutoEMation2 were used for Cryo-EM data collection with Titan Krios G3i (Thermo Fisher).

Data analysis: cryoSPARC v3.2.0, Relion 3.1.2, MotionCor2_v1.3.1-Cuda101, Phenix 1.18.2-3874-000, Coot 0.9.6, Pymol 2.4.0, pyem 0.5, UCSF ChimeraX-1.3, UCSF Chimera-1.15

For manuscripts utilizing custom algorithms or software that are central to the research but not yet described in published literature, software must be made available to editors and reviewers. We strongly encourage code deposition in a community repository (e.g. GitHub). See the Nature Portfolio guidelines for submitting code & software for further information.

Data

Policy information about availability of data

All manuscripts must include a data availability statement. This statement should provide the following information, where applicable:

- Accession codes, unique identifiers, or web links for publicly available datasets
- A description of any restrictions on data availability
- For clinical datasets or third party data, please ensure that the statement adheres to our policy

The cryo-EM density maps of the OGT dimer and the OGT-OGA complex generated in this study have been deposited to the Electron Microscopy Data Bank under the accession numbers EMD-33768 (OGT dimer), EMD-33767 (OGT–OGA conformer I), EMD-33773 (OGT–OGA conformer II), and EMD-33769 (OGT–OGA

conformer III). Atomic coordinates have been deposited to the RCSB Protein Data Bank under the accession numbers 7YEA (OGT dimer) and 7YEH (OGT-OGA conformer II). Source data are provided with this paper.

Research involving human participants, their data, or biological material

Policy information about studies with human participants or human data. See also policy information about sex, gender (identity/presentation), and sexual orientation and race, ethnicity and racism.

Reporting on sex and gender	N/A
Reporting on race, ethnicity, or other socially relevant groupings	N/A
Population characteristics	N/A
Recruitment	N/A
Ethics oversight	N/A

Note that full information on the approval of the study protocol must also be provided in the manuscript.

Field-specific reporting

Please select the one below that is the best fit for your research. If you are not sure, read the appropriate sections before making your selection.

☒ Life sciences ☐ Behavioural & social sciences ☐ Ecological, evolutionary & environmental sciences

For a reference copy of the document with all sections, see nature.com/documents/nr-reporting-summary-flat.pdf

Life sciences study design

All studies must disclose on these points even when the disclosure is negative.

Sample size	No statistic method was used to predetermine the sample size. Sample sizes were chosen based on standard practices in the field of research and previous analyses with similar experimental paradigms (See for example Meek et al, Nature Communications 2021, PMID: 34764280). The number of cryo-EM micrographs and particles for structural determination is shown in Supplementary Table 1.
Data exclusions	The exclusion criteria were not pre-established. 2D and 3D classification yielded multiple classes. Only the particles in the class that showed clear structural signals were selected, combined and used in the final reconstruction and refinement.
Replication	Multiple rounds of structural refinement have been performed and successful and all resulted in same density maps (with different resolutions though). Experiments of SDS-PAGE, WB analysis have been repeated for at least three times with similar results.
Randomization	Gold standard Fourier Shell Correlation method was used for cryo-EM 3D refinement, all particles were randomly split into two half groups. The process of splitting the dataset, odd and even, is considered random.
Blinding	The investigators were blinded to group allocation during cryo-EM data collection, processing and analysis. Blinding is not relevant for protein structure determination because these results are not subjective. Our procedure complies with the common practice in this field.

Reporting for specific materials, systems and methods

We require information from authors about some types of materials, experimental systems and methods used in many studies. Here, indicate whether each material, system or method listed is relevant to your study. If you are not sure if a list item applies to your research, read the appropriate section before selecting a response.

Materials & experimental systems

n/a	Involved in the study	
☐	☒	Antibodies
☐	☒	Eukaryotic cell lines
☒	☐	Palaeontology and archaeology
☒	☐	Animals and other organisms
☒	☐	Clinical data
☒	☐	Dual use research of concern
☒	☐	Plants

Methods

n/a	Involved in the study	
☒	☐	ChIP-seq
☒	☐	Flow cytometry
☒	☐	MRI-based neuroimaging

Antibodies

Antibodies used	rabbit anti-OGT (Abcam, ab177941), mouse anti-OGA (Abcam, ab68522), rabbit anti-OGA (Proteintech, 14711- 1-AP), mouse anti-Myc (Sigma-Aldrich, M4439), anti-Flag (Sigma-Aldrich, F1804), mouse anti-O-GlcNAc (Cell Signaling, CTD110.6, 9875s), mouse anti-O-GlcNAc (RL2) (Abcam, ab2739), and mouse anti-GAPDH (Proteintech, 60004-1-Ig).
Validation	All antibodies used in this research were purchased commercially or previously published. All primary antibodies have been validated by the manufacturers or the original developer.

Eukaryotic cell lines

Policy information about cell lines and Sex and Gender in Research

Cell line source(s)	HEK293FT cells
Authentication	Those obtained from ATCC were authenticated by morphological evaluation cross-checking with the description and images provided by the supplier.
Mycoplasma contamination	All cell lines were tested for mycoplasma contamination and tested negative.
Commonly misidentified lines (See ICLAC register)	No commonly misidentified cell lines were used.
