# Supplementary material for: Cryo-EM structure of human O-GlcNAcylation enzyme pair OGT-OGA complex
Source: Nat Commun. 2023 Oct 31;14:6952. doi: 10.1038/s41467-023-42427-8 (PMC10618255; doi:10.1038/s41467-023-42427-8)
Supplement: Supplementary file 4 — Description of Additional Supplementary Files [file 41467_2023_42427_MOESM4_ESM.pdf]

File name: Supplementary Movie 1

Description: Cryo-EM map and structure of human OGT homodimer is shown on the left with each domain individually colored. Cryo-EM map and structure of human OGT-OGA complex is shown on the right with each domain individually colored. The OGA N-terminal catalytic domain is shown in light green and its flexible region is shown in yellow.

File name: Supplementary Movie 2

Description: The conformational changes of OGT dimer upon OGA binding. The TPR domain becomes more rigid and compacted, and the distance between the two active sites in the dimer becomes shorter upon OGA binding. The OGT and OGA are individually colored. The OGA N-terminal catalytic domain is shown in light green and its flexible region is shown in yellow.
